# Supplementary material for: Sirtuin 6 Overexpression Improves Rotator Cuff Tendon-to-Bone Healing in the Aged
Source: Cells. 2023 Aug 10;12(16):2035. doi: 10.3390/cells12162035 (PMC10453227; doi:10.3390/cells12162035)
Supplement: Supplementary file 1 [file cells-12-02035-s001.zip › cells-2440683-supplementary/Supplemental material.pdf]

Sirtuin 6 overexpression improves rotator cuff tendon-to-bone healing in the aged

Contents

1. Supplemental figures

2. Supplemental table

1. Supplemental Figures

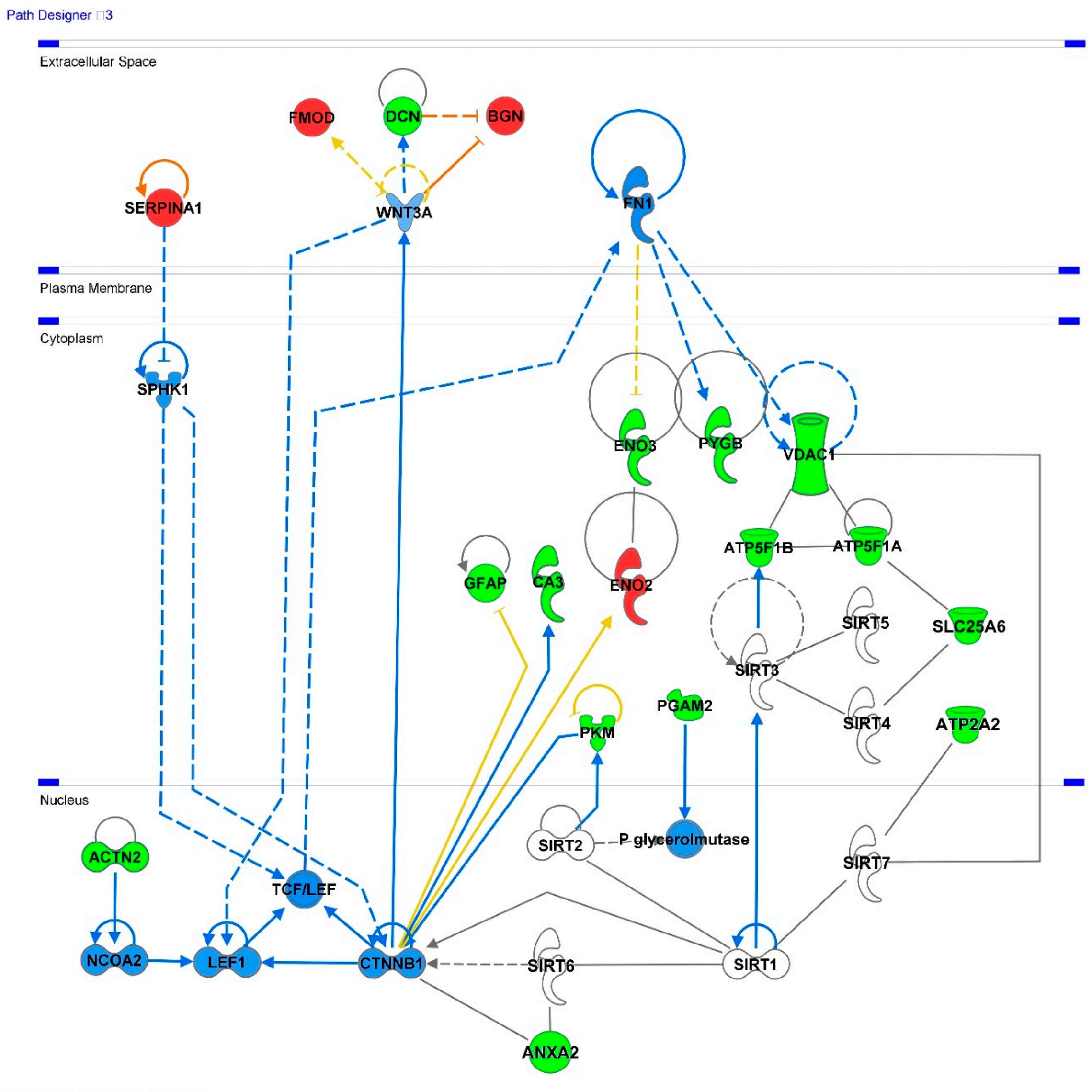

© 2000-2019 QIAGEN. All rights reserved.

Figure S1. The signaling network of sirtuins and Wnt-βcatenin signaling with age in rotator cuff proteome assessed by IPA network analysis (QIAGEN Inc.,). The molecular symbols in red and green indicate increased and decreased expression in the dataset, respectively. The molecular symbols in orange and blue indicate predicted activation and predicted inhibition in the dataset, respectively.

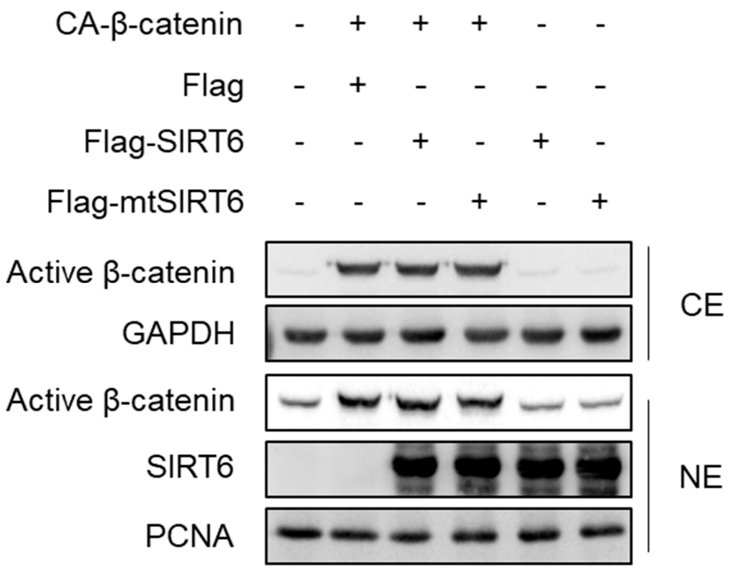

Figure S2. The level of active β-catenin according to various conditions. HEK293 cells were transfected with constitutive activation of β-catenin (CA-β-catenin) with SIRT6 or mutant SIRT6 (mtSIRT6), and protein levels of active β-catenin in cytosolic extract (CE) and nuclear extract (NE) were determined.

2. Supplemental Table S1

Table S1. Sequences and accession numbers for primers (forward, FOR; reverse, REV)

Gene	Sequences for primers	Accession No.
SIRT6	FOR: CCCACGGAGTCTGGACCAT	NM_001193285
	REV: CTCTGCCAGTTTGTCCCTG	
GAPDH	FOR: GGAGCGAGATCCCTCCAAAAT	NM_001256799.3
	REV: GGCTGTTGTCATACTTCTCATGG	
Ctnnb1	FOR: AAGTTCTTGGCTATTACGACA	XM_039082209.1
	REV: ACAGCACCTTCAGCACTCT	
Ccnd1	FOR: CCTCTCCTGCTACCGCACAA	XM_008760168.2
	REV: CGCAGGCTTGACTCCAGAAG	
Sost	FOR: ATGCAGCTCTCACTAG	NM_030584.2
	REV: TAGGCGCGGGCTCGCTCTA	
Dkk1	FOR: AATCGAGGAAGGCATCATTG	NM_001106350.1
	REV: CTGTGCTTGGTGCATACCTG	
Actb	FOR: AGCCATGTACGTAGC	NM_031144.3
	REV: TCTCCGGAGTCCATCACAATG	
